# Relapses of Anti-NMDAR, Anti-GABABR and Anti-LGI1 Encephalitis: A Retrospective Cohort Study

**DOI:** 10.3389/fimmu.2022.918396

**Published:** 2022-06-09

**Authors:** Rui Zhong, Qingling Chen, Xinyue Zhang, Hanyu Zhang, Weihong Lin

**Affiliations:** ^1^ Department of Neurology, The First Hospital of Jilin University, Changchun, China; ^2^ Department of Hepatology, Second People’s Clinical College of Tianjin Medical University, Tianjin, China

**Keywords:** relapse, immunotherapy delay, anti-NMDAR encephalitis, anti-GABABR encephalitis, anti-LGI1 encephalitis

## Abstract

**Objective:**

To investigate the relapse rate and study the factors that may predict the subsequent relapse in anti-NMDAR, anti-GABABR and anti-LGI1 encephalitis in Northeast China.

**Methods:**

In the retrospective cohort study, we consecutively enrolled patients with anti-N1MDAR, anti-GABABR and anti-LGI1 encephalitis between March 2015 and November 2021. The patients were followed up for at least 6 months. The outcome variable was a binary variable of relapse or not. Predictors of relapse were identified.

**Results:**

A total of 100 patients were enrolled. Relapse occurred in 26 (26%) patients after a median follow-up of 18 months since the first event. The relapse rates of anti - NMDAR, anti - GABABR and anti - LGI1 encephalitis were 25%, 33.3%, and 28.6%, respectively. The multivariable analysis results suggested that immunotherapy delay at the acute phase was independently associated with an increased risk of relapse in total patients (HR = 2.447, 95% CI = 1.027 - 5.832; P = 0.043). Subgroup analysis results showed that antibody titer was associated with the likelihood of relapse in anti-LGI1 encephalitis. The higher the concentration, the more likely it was for patients to have relapse (p=0.019).

**Conclusion:**

The general relapse rate of anti-NMDAR, anti-GABABR and anti-LGI1 encephalitis was 26%. The risk of subsequent relapse was elevated in those with delayed immunotherapy in the first episode. In subgroup of anti-LGI1 encephalitis, higher antibody titer was the risk factors of relapse. Thus, timely and aggressive immunotherapy may be beneficial for patients to prevent subsequent relapse.

## Introduction

Autoimmune encephalitis (AE) comprises a group of potentially life-threatening autoimmune disorders of the brain parenchyma, which was associated with auto-antibodies against surface receptors and ion channels on neurological tissues (1–3). Although some AE patients developed critical illness and faced a life-threatening condition in the acute stage, most of them achieved favorable long-term functional outcomes after receiving immunotherapy (4–6).

According to the literature, some AE patients may have one or multiple relapse after the first event (7–9). 8 - 36.4% of patients with anti-NMDAR encephalitis may relapse (7, 9). And relapses occurred in about 14 - 35% of anti-LGI1 encephalitis (10, 11). The relapse rates varied across studies, indicating that AE is heterogeneous among people of different races. Regarding the predictors of relapse, a recent cohort study from Western China suggested that female sex and delayed immunotherapy were relapse-related risk factors in anti-NMDAR encephalitis (9). Sleep disorders at the acute phase may increase the risk of relapse in anti - LGI1 encephalitis (11). The use of immunotherapy in the initial episode was related with a lower likelihood of relapses in anti-NMDAR encephalitis, and a relapse-decreasing effect of second-line immunotherapy was also reported (4).

To date, large cohort studies on the relapse rate and predictors of relapse in anti-NMDAR, anti-GABABR and anti-LGI1 encephalitis are lacking in Northeast China. We retrieved information from a tertiary university hospital in Northeast China for this retrospective cohort study. Thus, we focused on patient relapse rate and also identified factors that may predict the subsequent relapse in anti-NMDAR, anti-GABABR and anti-LGI1 encephalitis.

## Methods

### Patients and Study Design

The retrospective cohort study was performed at a tertiary university hospital serving a population of more than 5,000,000 each year in Northeast China. In the current study, we consecutively recruited patients with definitive diagnosis of anti-NMDAR, anti-GABABR and anti-LGI1 encephalitis who were hospitalized at the Department of Neurology, Jilin University First Hospital between March 2015 and November 2021. The definitive diagnosis was established by at least two neurologists according to the definitions of AE from a consensus statement proposed in 2016 (2). Auto-antibodies were detected in cerebrospinal fluid (CSF) and/or serum samples using cell-based assays. Tumor was screened using whole body PET/CT, chest and abdomen CT and/or other methods. The exclusion criteria were as follows: (1) patients with clinical and laboratory evidence of CNS viral, bacterial, fungal, parasitic, or mycobacterium tuberculosis infection; (2) patients with other severe neurological diseases such as Parkinson’s disease, stroke, or acute demyelinating encephalomyelitis; (3) patients with positive results for other type of AE auto-antibodies or neurological paraneoplastic antibodies detected in CSF and/or serum samples; (4) patients with a follow-up period shorter than 6 months. This study was approved by the ethics committee of the First Hospital of Jilin University, which also waived the requirement for written informed consent due to the non-interventional design according to national regulations.

### Data Collection

Observational data of patients at the acute phase were retrospectively collected from our hospital electronic medical records, and face to face interviews were performed by experienced clinicians. The detailed data collection consisted of demographic information, presenting symptoms, hospitalization data, ancillary examination, immunotherapy and relapse. The decisions about the choice of immunotherapy and treatment duration were based on physicians’ experience.

### Definitions

We defined status epilepticus (SE) as a continuous seizure activity lasting longer than 5 minutes (12). We defined patients with immunotherapy delay as the subjects who start an immunotherapy regimen at least 28 days after the disease onset (13, 14). A hospital length of stay >30 days was defined as long hospital stay. Relapse of AE was defined as new onset or worsening of symptoms after an initial improvement or stabilization of at least 2 months (4).

### Follow-up and Outcome Assessment

Patients were followed up by their treating physician *via* telephone interviews and/or clinical visits. The outcome variable was a binary variable of relapse or not. Formal follow-up outcome assessments were undertaken every 6 months after the disease onset. At each follow-up or clinical visit, patients and their relatives were questioned regarding patient’s general conditions and complaints. Patients were also asked to contact their treating physician within days of experiencing a possible relapse (new onset or worsening of symptoms). Follow-up ended when the patient died or was lost to follow-up.

### Statistical Analysis

SPSS version 26.0 (SPSS, Chicago, IL, USA) was used for all data analysis. Continuous variables were described as medians (interquartile ranges [IQR]), and categorical variables were described as counts (percentages). Univariate analysis of continuous variables were performed using Mann - Whitney U test. While Pearson’s chi - squared or Fisher’s exact test were used for categorical variables where applicable. Variables with p < 0.05 in univariate analysis were retained in multivariate analysis. Independent predictors of clinical relapse were estimated using a Cox regression model. Hazard ratios (HRs) in the cox model and corresponding 95% confidence interval (CI) were conducted to evaluate the strength of association. The P value of <0.05 was considered significant.

## Results

### Patient Characteristics

The medical records of 110 hospitalized patients were reviewed. A total of 100 patients were enrolled based on the inclusion criteria. This cohort comprised of 40 patients with anti-NMDAR encephalitis, 24 with anti-GABABR encephalitis, and 36 with anti-LGI1 encephalitis. The patient demographic and clinical characteristics were described in **Table 1**. The median age at disease onset was 52 years (interquartilerange [IQR]33 - 66), and 54 patients (54%) were male. After a median follow-up of 18 months (IQR 10- 30) since the first event, relapses were diagnosed in 26 patients (26.0%). The median duration from onset to the first relapse was 10 (range: 2–29) months. During the first 24 months, 23 (88.5%) patients experienced an initial relapse. 3 patients (3%) had 2 or more relapse events.

**Table 1:** Demographic and clinical characteristics of the first episode in the total cases, relapsing, and non - relapsing patients.

Variables	Total cases (n=100)	Relapsing (n=26)	No relapsing (n=74)	p Value
Age at onset (years)	52 (33, 63)	57 (38, 66)	48 (33, 62)	0.225
Gender - Male	54 (54.0)	12 (46.2)	42 (56.8)	0.351
Presenting symptom				
Fever ( > 37.5°C)	34 (34.0)	8 (30.8)	26 (35.1)	0.686
Acute symptomatic seizure	87 (87.0)	24 (92.3)	63 (85.1)	0.505
Status epilepticus	42 (42.0)	9 (34.6)	33 (44.6)	0.375
Psychiatric symptoms	64 (64.0)	18 (69.2)	46 (62.2)	0.518
Movement disorders	37 (37.0)	7 (26.9)	30 (40.5)	0.216
Cognitive impairment	65 (65.0)	18 (69.2)	47 (63.5)	0.599
Speech disturbance	19 (19.0)	6 (23.1)	13 (17.6)	0.567
Impairment of consciousness	36 (36.0)	9 (34.6)	27 (36.5)	0.864
Sleep disorders	39 (39.0)	9 (34.6)	30 (40.5)	0.594
Tumor	11 (11.0)	2 (7.7)	9 (12.2)	0.723
Ancillary examination				
Abnormal EEG	57 (57.0)	16 (61.5)	41 (55.4)	0.587
Abnormal brain MRI	55 (55.0)	12 (46.2)	43 (58.1)	0.292
Abnormal CSF	86 (86.0)	22 (84.6)	64 (86.5)	0.754
Antibodies type				
NMDAR	40 (40.0)	10 (38.5)	30 (40.5)	0.619
GABABR	24 (24.0)	8 (30.8)	16 (21.6)	
LGI1	36 (36.0)	8 (30.8)	28 (37.8)	
Antibody titer				
+	31 (31.0)	8 (30.8)	23 (31.1)	0.433
++	51 (51.0)	11 (42.3)	40 (54.1)	
+++	18 (18.0)	7 (26.9)	11 (14.9)	
Treatment				
First-line immunotherapy				
MTP	85 (85.0)	22 (84.6)	63 (85.1)	1
IVIG	76 (76.1)	21 (80.8)	55 (74.3)	0.508
MTP + IVIG	62 (62.0)	17 (65.4)	45 (60.8)	0.679
Second-line immunotherapy	6 (6.0)	1 (3.8)	5 (6.8)	1
Without immunotherapy	1 (1.0)	0 (0)	1 (1.4)	1
Immunotherapy delay	50 (50.0)	18 (69.2)	32 (43.2)	**0.023**
ICU admission	45 (45.0)	11 (42.3)	34 (45.9)	0.748
Long hospital stay	31 (31.0)	9 (34.6)	22 (29.7)	0.643
Follow-up duration (Months)	18 (10, 30)	18 (11, 30)	18 (9, 28)	0.877

EEG, electroencephalogram; MRI, magnetic resonance imaging; CSF, cerebrospinal fluid; NMDAR, N-methyl-D-aspartate receptor; LGI1, leucin-rich glioma inactivated-1; GABABR, g-aminobutyric acid type B receptor; MTP, methylprednisolone; IVIG, intravenous immunoglobulin; ICU, intensive care unit.

Bold entries indicate p < 0.05.

### Univariate Analysis of Predictors of Relapse

Comparison of demographic and clinical characteristics between the relapsing and non-relapsing patients were also summarized in **Table 1**. Compared with the no relapse group, patients in relapse group were more prone to receive delayed immunotherapy (p = 0.023). The relapse rates did not differ significantly between anti-NMDAR, anti-GABABR and anti-LGI1 encephalitis (p = 0.619). No significant differences were observed between the first episode of relapsing and non-relapsing patients in terms of other variables (p > 0.05).

### Multivariable Analysis of Predictors of Relapse

**Figure 1:**
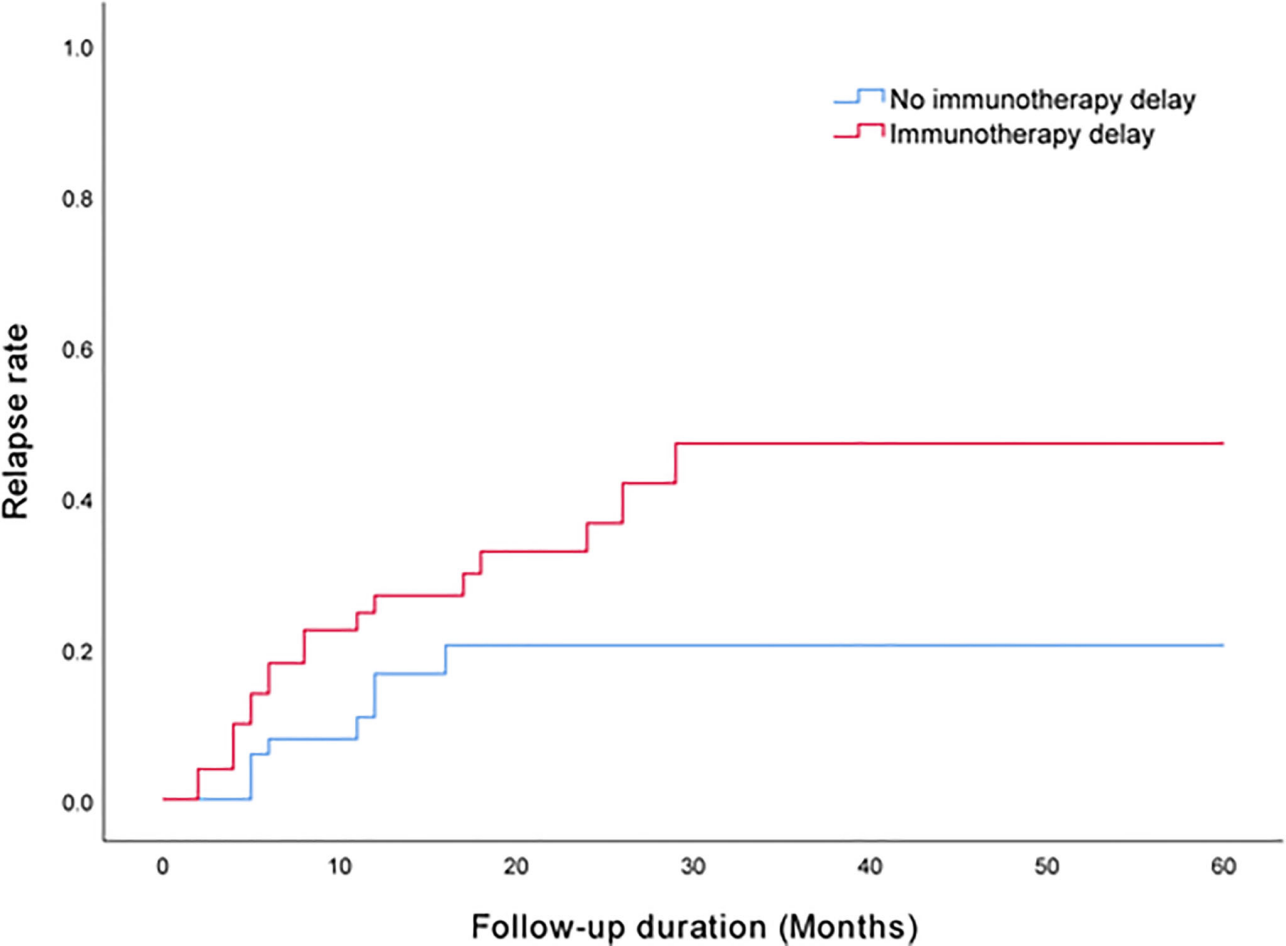
Kaplan - Meier curve shows that immunotherapy delay was associated with an increased risk of relapse.

A univariate analysis was performed for preliminary identification of variables associated with clinical relapse. Immunotherapy delay was identified at a significance level of p < 0.05 (**Table 1**). According to prior literature, gender (9), sleep disorders (11), tumor (15), abnormal brain MRI (7) and second-line immunotherapy (4) may be related to clinical relapse. Accordingly, these variables were retained for the multivariable cox regression model, despite p values > 0.05. The HR of patients treated with a delayed immunotherapy experiencing relapse were 2.447 times greater than those of patients treated with a timely immunotherapy (HR = 2.447, 95% CI = 1.027 - 5.832; P = 0.043). The results are described in **Table 2** and **Figure 1**.

**Table 2:** Multivariable analysis of predictors of clinical relapse.

Variables	Hazard Ratios	95% CI	p Value
Gender			
Male	0.774	0.346 - 1.734	0.534
Female	Ref		
Sleep disorders			
Yes	0.955	0.417 - 2.187	0.914
No	Ref		
Tumor			
Yes	0.948	0.215 - 4.179	0.944
No	Ref		
Abnormal brain MRI			
Yes	0.56	0.251 - 1.245	0.155
No	Ref		
Second-line immunotherapy			
Yes	0.867	0.114 - 6.559	0.89
No	Ref		
Immunotherapy delay			
Yes	2.447	1.027 - 5.832	**0.043**
No	Ref		

MRI, magnetic resonance imaging; CI, confidence interval.

Bold entries indicate p < 0.05.

### Subgroup Analysis

Comparisons between relapse group and no relapse group in each encephalitis are summarized in **Table 3**. In anti-NMDAR encephalitis, subgroup analysis also revealed a significant association between subsequent relapse and immunotherapy delay at the acute phase(p=0.018). As for patients with anti-GABABR encephalitis, no factor was associated with relapse, probably due to the limited sample size. In anti-LGI1 encephalitis, antibody titer was associated with the likelihood of relapse. The higher the concentration, the more likely it was for patients to have subsequent relapse (p=0.019). The results are described in **Table 3**.

**Table 3:** Univariate analysis of variables associated with relapse in anti-NMDAR, anti-GABABR and anti-LGI1 encephalitis, respectively.

Variables	NMDAR (n=40)			GABABR (n=24)			LGI1 (n=36)		
	Relapsing (n=10)	No relapsing (n=30)	p Value	Relapsing (n=8)	No relapsing (n=16)	p Value	Relapsing (n=8)	No relapsing (n=28)	p Value
Age at onset (years)	30 (21, 53)	32 (21, 39)	0.612	59 (56, 63)	63 (54, 66)	0.383	66 (57, 72)	56 (48, 65)	0.099
Gender - Male	6 (60.0)	16 (53.3)	1	4 (50.0)	9 (56.3)	1	2 (25.0)	17 (60.7)	0.114
Presenting symptom									
Fever (>37.5°C)	5 (50.0)	16 (53.3)	1	3 (37.5)	5 (31.3)	1	0 (0)	5 (17.9)	0.566
Acute symptomatic seizure	8 (80.0)	25 (83.3)	1	8 (100.0)	14 (87.5)	0.536	8 (100.0)	24 (85.7)	0.555
Status epilepticus	2 (20.0)	13 (43.3)	0.269	6 (75.0)	10 (62.5)	0.667	1 (12.5)	10 (35.7)	0.388
Psychiatric symptoms	8 (80.0)	21 (70.0)	0.696	7 (87.5)	9 (56.3)	0.189	3 (37.5)	16 (57.1)	0.434
Movement disorders	5 (50.0)	18 (60.0)	0.717	1 (12.5)	3 (18.8)	1	1 (12.5)	9 (32.1)	0.397
Cognitive impairment	8 (80.0)	17 (56.7)	0.269	5 (62.5)	6 (37.5)	0.39	5 (62.5)	24 (85.7)	0.167
Speech disturbance	4 (40.0)	8 (26.7)	0.451	1 (12.5)	3 (18.8)	1	1 (12.5)	2 (7.1)	0.541
Impairment of consciousness	4 (40.0)	17 (56.7)	0.473	4 (50.0)	6 (37.5)	0.673	1 (12.5)	4 (14.3)	1
Sleep disorders	3 (30.0)	13 (43.3)	0.711	3 (37.5)	6 (37.5)	1	3 (37.5)	11 (39.3)	1
Tumor	0	5 (16.7)	0.306	1 (12.5)	4 (25.0)	0.631	1 (12.5)	0 (0)	0.222
Ancillary examination									
Abnormal EEG	4 (40.0)	15 (50.0)	0.721	7 (87.5)	9 (56.3)	0.189	5 (62.5)	17 (60.7)	1
Abnormal brain MRI	3 (30.0)	17 (56.7)	0.273	4 (50.0)	6 (37.5)	0.673	5 (62.5)	21 (75.0)	0.658
Abnormal CSF	8 (80.0)	28 (93.3)	0.256	8 (100.0)	14 (87.5)	0.536	6 (75.0)	22 (78.6)	1
Antibody titer									
+	5 (50.0)	12 (40.0)	0.899	2 (25.0)	2 (12.5)	0.27	1 (12.5)	9 (32.1)	**0.019**
++	3 (30.0)	14 (46.7)		5 (62.5)	9 (56.3)		3 (37.5)	17 (60.7)	
+++	2 (20.0)	4 (13.3)		1 (12.5)	5 (31.3)		4 (50.0)	2 (7.1)	
Treatment									
First-line immunotherapy									
MTP	8 (80.0)	27 (90.0)	0.584	7 (87.5)	12 (75.0)	0.631	7 (87.5)	24 (85.7)	1
IVIG	10 (100.0)	27 (90.0)	0.56	6 (75.0)	9 (56.3)	0.657	5 (62.5)	19 (67.9)	1
MTP + IVIG	8 (80.0)	24 (80.0)	1	5 (62.5)	6 (37.5)	0.39	4 (50.0)	15 (53.6)	1
Second-line immunotherapy	0	4 (13.3)	0.556	0	0	_	1 (12.5)	1 (3.6)	0.4
Without immunotherapy	0	0	_	0	1 (6.3)	1	0 (0)	0 (0)	_
Immunotherapy delay	7 (70.0)	7 (23.3)	**0.018**	4 (50.0)	6 (37.5)	0.673	7 (87.5)	19 (67.9)	0.397
ICU admission	6 (60.0)	18 (60.0)	1	5 (62.5)	11 (68.8)	1	0 (0)	5 (17.9)	0.566
Long hospital stay	5 (50.0)	19 (63.3)	0.482	2 (25.0)	1 (6.3)	0.249	2 (25.0)	2 (7.1)	0.207
Follow-up duration (Months)	25 (12, 32)	12 (6, 23)	0.077	6 (6, 12)	12 (6, 30)	0.106	24 (18, 45)	24 (13, 36)	0.614

EEG, electroencephalogram; MRI, magnetic resonance imaging; CSF, cerebrospinal fluid; NMDAR, N-methyl-D-aspartate receptor; GABABR, g-aminobutyric acid type B receptor; LGI1, leucin-rich glioma inactivated-1; MTP, methylprednisolone; IVIG, intravenous immunoglobulin; ICU, intensive care unit.

Bold entries indicate p < 0.05.

## Discussion

The findings of our study provided some relevant insights regarding the relapse rate and relapse-related risk factors in anti-NMDAR, anti-GABABR and anti-LGI1 encephalitis. The main findings are as follows:(1) 26% of patients had one or multiple relapse during a median follow-up of 18 months. The relapse rates of anti - NMDAR, anti - GABABR and anti - LGI1 encephalitis were 25%, 33.3%, and 28.6%, respectively. (2) Immunotherapy delay predicts an increased risk of relapse. Additionally, Subgroup analysis suggested that higher antibody titer was the risk factor for relapse in anti - LGI1 encephalitis. Note that the same population is used in both the current manuscript and the published article (16). Different from the published article that focused on mortality in AE (16), the current manuscript investigated the relapse rate and studied the factors that may predict the subsequent relapse in the same population.

Regarding the relapse rates, a recent large cohort study from Western China suggested that 15.9% of anti - NMDAR encephalitis patients had one or multiple relapses, which is relative lower than that found in our cohort (25.0%) (9). The relapse rate reported in prior literature varied between 8% and 25% in anti - NMDAR encephalitis, even reaching 36.4% (8, 17, 18). The varying relapse rates may be partly explained by the differences in follow-up period, genetic backgrounds or the etiology of the disease (8). Additionally, 28.6% of anti - LGI1 encephalitis patients had relapse in this cohort, which is comparable with the previously reported incidences of 14 - 35% (19–22).

The presence of tumor was observed in about 10-15% of anti - NMDAR encephalitis patients in Mainland China (9, 16, 23), comparable with 20 - 59% reported by prior literature in Western countries (4, 24). The relationship between the presence of tumor and subsequent relapse remains unclear. Dalmau et al. reported that the risk of further relapse was higher in patients without tumor (15). However, this finding is not in line with the recent reports that found the presence of tumor and tumor removal were both not associated with subsequent relapse (7, 8, 25). In a recent cohort study from Central China, abnormal brain MRI has been identified as a risk factor for relapse in anti - NMDAR encephalitis (25). Similarly, a brainstem lesion on MRI was also reported to be associated with a greater chance of relapse (7). However, this association was not supported by our data and other literature (4, 8, 11). It may be due to the difference in patient characteristics and sample size across studies.

Regarding the treatment modalities, patients who did not receive immunotherapy in the initial episode had an increased risk of relapse (4, 8, 26). In a cohort of anti - NMDAR encephalitis, patients who were treated with second-line immunotherapy during relapses also had a lower risk for relapses (4). Consistently, the chance of further relapse was grater in patients who did not receive aggressive immunotherapy (27). However, we did not observe the relationship between second-line immunotherapy during the initial episode and the risk of relapse in this study. It may be because only small number of patients (n=6, 6%) used second-line immunotherapy in our cohort. Our study showed that a delayed immunotherapy increased the risk of subsequent relapses, which was in line with other studies (4, 9). Thus, timely immunotherapy may be beneficial for patients to prevent subsequent relapses. In other antibody-mediated autoimmune diseases such as myasthenia gravis (MG), similar beneficial effect of early immunosuppressive treatment on preventing disease progression has also been established (28). Considering the limited sample size, associations between relapse and different immunotherapy strategies require more extensive cohort studies to verify.

In subgroup analysis, we discovered that antibody titer was associated with the likelihood of relapse in anti-LGI1 encephalitis. The higher the concentration, the more likely it was for patients to have relapse. Of interest, it has recently been reported that higher CSF IgG4 subclass‐specific titers strongly correlated with worse outcome in anti-LGI1 encephalitis (29). Consistently, in a cohort of anti - NMDAR encephalitis patients, higher antibody titer in CSF and serum seemed to increase the risk of poor clinical outcome (17), confirming in part the findings of another study (30). Additionally, it was worth noting that the antibody titer change in CSF was more closely related with relapses than was that in serum in anti - NMDAR encephalitis (17). Our finding suggested that CSF antibody titer indices warrant further evaluation as a prognostic factor in LGI1 autoimmunity. A higher antibody titer might indicate the need for more aggressive initial immunotherapy. Further studies are needed to confirm our finding.

The study has some limitations. First, the patients were enrolled from a single center and the sample size was relative small. Thus, there may exist selection bias. Second, the follow-up period is relatively short and we cannot rule out that patients in the no relapse group may have relapse in the future. Thus, The relapse rate may be underestimated. Third, The use of immunotherapy was not associated with the subsequent relapses in our study. It may be because only one patient did not receive immunotherapy, which could limit the statistical power. Furthermore, the number of patients received second-line immunotherapy was small. Further studies are warranted to evaluate the efficacy of second line immunotherapy on relapse. Fourth, we adjusted for many possible predictors of relapse in the multivariable model, but the possibility of residual confounding remains such as cancer treatment. Finally, multivariable analyses of the predictors of relapse could not be performed due to the limited cases in each encephalitis subgroup.

In conclusion, we observed relapses in 26% of patients after a median follow-up of 18 months. The risk of relapse was elevated in those with delayed immunotherapy in the first episode. Thus, timely immunotherapy may be beneficial for AE patients to prevent subsequent relapse. In subgroup of anti-LGI1 encephalitis, higher antibody titer was the risk factors of relapse.

## Data Availability Statement

The raw data supporting the conclusions of this article will be made available by the authors, without undue reservation.

## Ethics Statement

The studies involving human participants were reviewed and approved by Ethics Committee of the First Hospital of Jilin University. Written informed consent for participation was not provided by the participants’ legal guardians/next of kin because: The hospital ethics committee waived the requirement for written informed consent due to the non-interventional design according to national regulations.

## Author Contributions

WL and RZ conceived and designed the study. RZ, HZ, and XZ were involved in data acquisition. RZ and QC analyzed the data and wrote the manuscript. All authors contributed to the article and approved the submitted version.

## Conflict of Interest

The authors declare that the research was conducted in the absence of any commercial or financial relationships that could be construed as a potential conflict of interest.

## Publisher’s Note

All claims expressed in this article are solely those of the authors and do not necessarily represent those of their affiliated organizations, or those of the publisher, the editors and the reviewers. Any product that may be evaluated in this article, or claim that may be made by its manufacturer, is not guaranteed or endorsed by the publisher.
